# Therapeutic Potential of Sol–Gel ZnO Nanocrystals: Anticancer, Antioxidant, and Antimicrobial Tri-Action

**DOI:** 10.1021/acsomega.3c07191

**Published:** 2024-03-20

**Authors:** Busra Eren, Meliha Koldemir Gunduz, Gullu Kaymak, Derya Berikten, Zehra Banu Bahsi

**Affiliations:** † Institute of Biotechnology, Gebze Technical University, Gebze, Kocaeli 41400, Turkey; ‡ Faculty of Engineering and Natural Sciences, Department of Basic Sciences of Engineering, Kutahya Health Sciences University, Kütahya 43100, Turkey; § Training and Research Center, Kutahya Health Sciences University, Kütahya 43500, Turkey; ∥ Faculty of Engineering and Natural Sciences, Department of Molecular Biology and Genetics, Kütahya Health Sciences University, Kütahya 43100, Turkey

## Abstract

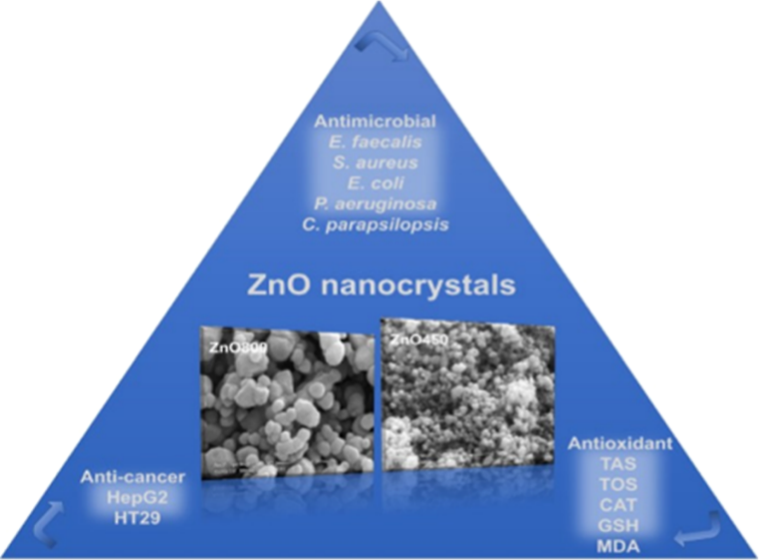

Zinc oxide nanocrystals (ZnO NCs) hold great promise in nanomedicine with fascinating multifunctional properties. We investigated the therapeutic potential of sol–gel synthesized ZnO NCs with crystal sizes of 52.65 and 25.11 nm, focusing on their anticancer effects on HepG2 and HT29 cells, antioxidant properties, and antimicrobial activity. Both samples displayed a hexagonal wurtzite ZnO structure, wherein the crystal sizes diminished with lower calcination temperatures according to X-ray diffraction. The scanning electron microscopy analysis revealed that lowering the calcination temperature resulted in a decrease in the grain size of the ZnO NCs, as expected. This reduction in grain size combined with a decrease in crystal size resulted in a significant 40% reduction in the reflectance of the ZnO NCs in UV–vis–NIR spectroscopy. It was also observed that the ZnO NCs calcined at higher temperatures exhibited larger particle sizes with a reduced surface area mean of 69.30 μm and a stable negative zeta potential of −11.2 mV. In contrast, the ZnO NCs calcined at lower temperatures exhibited a larger surface area mean of 34.56 μm and a positive zeta potential of +10 mV. In both cell lines, the cytotoxic potential was found to be higher in HepG2 cells. Specifically, when ZnO nanocrystals (NCs) with a crystal size of 52.65 nm were used, the lowest cell viability was observed at a concentration of 5.74 μg/mL. Based on oxidative stress index values, a lower crystal size of ZnO NCs displayed greater effectiveness in HT29 cells, while a higher crystal size of ZnO NCs had pronounced effects in HepG2 cells. Moreover, both ZnO NCs exhibited significant antimicrobial activity against Gram-positive bacteria (*Enterococcus faecalis* and *Staphylococcus aureus*) and *Candida parapsilopsis* fungus. These findings emphasize sol–gel ZnO NCs’ potential as versatile agents in nanomedicine, spurring research on targeted cancer therapies and antimicrobial innovations.

## 1 Introduction

Recent advances in nanotechnology have paved the way for the development of various nanomaterials with different therapeutic applications.^1^ Among these materials, zinc oxide nanoparticles (ZnO nanoparticles) are extremely versatile and are considered one of the most widely used candidates in various fields such as medicine, cosmetics, electronics, and textiles. They are particularly known for their usefulness in drug delivery and cancer diagnosis and treatment, utilizing their unique physical and chemical properties.^2−4^ The U.S. Food and Drug Administration (FDA) has classified ZnO nanoparticles as “Generally Recognized as Safe” (GRAS), indicating their biocompatibility, nontoxicity, and environmental safety for human use. They are characterized by a wide band gap of 3.37 eV and an exciton binding energy of 60 meV, which give them strong catalytic activity, UV protection, anti-inflammatory, and wound healing properties. Their antimicrobial efficacy extends to a wide range of pathogens, including bacteria, fungi, and viruses, making them a viable option for wound healing and infection management. In addition, ZnO nanoparticles possess strong antioxidant capabilities, scavenging free radicals, and reducing cellular oxidative stress, which is beneficial for the prevention or mitigation of diseases associated with oxidative damage. Zinc oxide nanoparticles (ZnO nanoparticles) are interesting for biomedical imaging due to their luminescent properties. They are also of interest for biosensing as they act as nanocarriers for a range of payloads such as drugs, genes, proteins, and imaging agents and exhibit pH-sensitive properties that are beneficial for targeted drug delivery to tumors. The ability of ZnO nanoparticles to induce the production of reactive oxygen species (ROS) highlights their potential as anticancer agents and offers a promising approach to eradicate cancer cells.^4^ The combination of anticancer, antimicrobial, and antioxidant properties in a single type of nanoparticle such as ZnO highlights their potential as a versatile tool in advanced medical treatments and diagnostics.

Common methods for preparing ZnO nanoparticles include sol–gel synthesis, precipitation, hydrothermal reaction, chemical vapor deposition, and thermal decomposition.^5,6^ Among these methods, sol–gel synthesis stands out as a particularly advantageous technique due to its simplicity, cost-effectiveness, and ability to achieve precise control over the size and morphology of ZnO nanoparticles. Additionally, sol–gel synthesis offers the potential for enhanced purity and the incorporation of dopants or functionalization for tailored properties in various applications.^2,7^ This synthesis method not only offers the possibility of producing high-quality materials of the same size on an industrial scale,^8^ but also corresponds to the current trend toward more sustainable and environmentally friendly manufacturing processes through integrated green synthesis or the use of sustainable materials.^9,10^

In cancer therapy, a key challenge is to overcome the resistance of tumors to conventional treatments while minimizing the harmful side effects.^4,11,12^ Therefore, the targeted and efficient use of ZnO nanoparticles is of great importance. In view of the increasing antimicrobial resistance of bacteria to existing antibiotics, ZnO nanoparticles also have the potential to meet a crucial need in the healthcare sector as an innovative and effective antimicrobial agent.^13^ ZnO nanoparticles have shown promise for a variety of therapeutic applications, but their size and concentration need to be carefully controlled to ensure both their efficacy and safety.^7,14^ While smaller nanoparticles may be more effective due to their increased surface-area-to-volume ratio, they can also be more prone to aggregation and have increased toxicity if not properly controlled. Additionally, higher concentrations can lead to toxic effects, including oxidative stress and inflammation.^15−19^ Therefore, understanding the precise mechanisms underlying the size-dependent effects of ZnO nanoparticles as well as the concentration levels of these nanoparticles is critical for developing safe and effective therapeutic applications. Further research is needed to fully elucidate the mechanisms of action and potential clinical applications of these nanoparticles.

The cancer cells HepG2 and HT29, which originate from human hepatocellular carcinoma and adenocarcinoma of the colon respectively, play a central role in cancer research as they represent two of the most common types of human cancer.^20−22^ The efficacy of ZnO nanoparticles in combating these cell lines is the subject of studies. For example, research on HepG2 (liver cancer) cells has shown that the cytotoxicity of ZnO nanoparticles depends on both the particle size and concentration. This study has shown that ZnO nanoparticles induce cell death in HepG2 cells primarily through necrosis caused by the release of Zn^2+^ ions and the induction of oxidative stress.^23^ In particular, Hassan et al. performed in vitro and in vivo studies with ZnO nanoparticles, which revealed their promising anticancer potential in various cancer cell lines, including human hepatocellular carcinoma (HEPG2), with an observed IC_50_ value of 33.11 μmol/L.^24^ In the context of HT29 cells, the cytotoxic effect of ZnO quantum dot nanoparticles (QD nanoparticles) with an IC_50_ value of 40 μg/mL was observed after 48 h of treatment, highlighting their concentration-dependent cytotoxic effect.^25^

The antibacterial properties of ZnO nanoparticles have also been the subject of research. Studies have shown that ZnO particles with hierarchical structures, such as tetrapods or flower-like formations, exhibit varying degrees of efficacy against bacterial strains such as *Escherichia coli* and *Staphylococcus aureus*. The antibacterial activity of these nano- and microstructures was found to be concentration-dependent, with different structural formations of ZnO exhibiting different levels of efficacy. Sharmila et al. biosynthesized spherical ZnO nanoparticles measuring 70–75 nm and demonstrated their effectiveness against both Gram-positive (*Bacillus subtilis* and *S. aureus*) and Gram-negative bacteria (*E. coli* and *Pseudomonas aeruginosa*).^26^ Gonzalez et al. synthesized ZnO nanoparticles in different morphologies—spherical, hexagonal, and rod-shaped—to study their antibacterial and anticancer properties. They found that spherical ZnO nanoparticles, with an average diameter of 20 ± 4 nm, were most effective in inhibiting *E. coli, S. aureus*, and *HeLa cells*, especially at a concentration of 100 μg/mL. This study emphasizes the crucial role of nanoparticle size and shape in determining biological activity, with smaller spherical nanoparticles showing higher efficacy.^27^ In addition, ZnO nanoparticles were used as carriers for the encapsulation of the cancer drug 5-fluorouracil. This innovative approach not only facilitates the administration of the drug but also enhances its antitumor activity, as demonstrated by the increased toxicity to MCF-7 cells. Such encapsulation strategies represent a promising direction for comprehensive cancer treatment by combining the therapeutic effects of ZnO nanoparticles and anticancer drugs.^28^ Overall, these studies show the significant potential of ZnO nanoparticles for medical applications, especially for antimicrobial and anticancer therapies, and emphasize the importance of nanoparticle properties such as size, shape, and surface modification for improving their efficacy.

In this research study, we report the synthesis of sol–gel ZnO nanocrystals (NCs) and their characterization using different techniques. We then investigate their therapeutic potential as a function of their crystal size and concentration, including anticancer activity on HepG2 and HT29 cancer cells, antioxidant properties, and antimicrobial activity. The therapeutic potential of sol–gel synthesized ZnO NCs with tri-action, combining anticancer effects on specific cancer cells, antioxidant capabilities, and antimicrobial properties, represents a unique and innovative approach in the field of medical research. In particular, the anticancer properties of ZnO nanoparticles against HepG2 and HT29 cancer cells have shown promise for the development of new cancer therapies. The multifunctional nature of these NCs sets them apart as a novel and promising avenue for targeted therapy and overall treatment efficacy. ZnO NCs have been widely studied for their potential applications in various fields, including biomedical and material sciences. However, the specific findings and conclusions derived from this investigation can still contribute to the existing body of knowledge in these areas and provide valuable insights into the development of novel nanomaterials for cancer therapy and antimicrobial applications.

## 2 Materials and Methods

### 2.1 Materials

All chemicals were obtained from Sigma-Aldrich and used without further purification. Zinc acetate dihydrate (Zn(CH_3_COO)_2_·2H_2_O) with 99% purity was preferred as the zinc precursor. Sodium hydroxide (NaOH) was used to adjust the pH to 12 as a source of hydroxyl groups. Methanol (CH_3_OH) with (99%) and deionized water (H_2_O) were used as solvents. Methanol and sodium hydroxide help to prepare a stoichiometric solution for obtaining ZnO NCs, as well as adjusting the homogeneity and pH value of the solution.^6^

### 2.2 Synthesis of ZnO NCs Using Sol–gel Method

**Figure 1:**
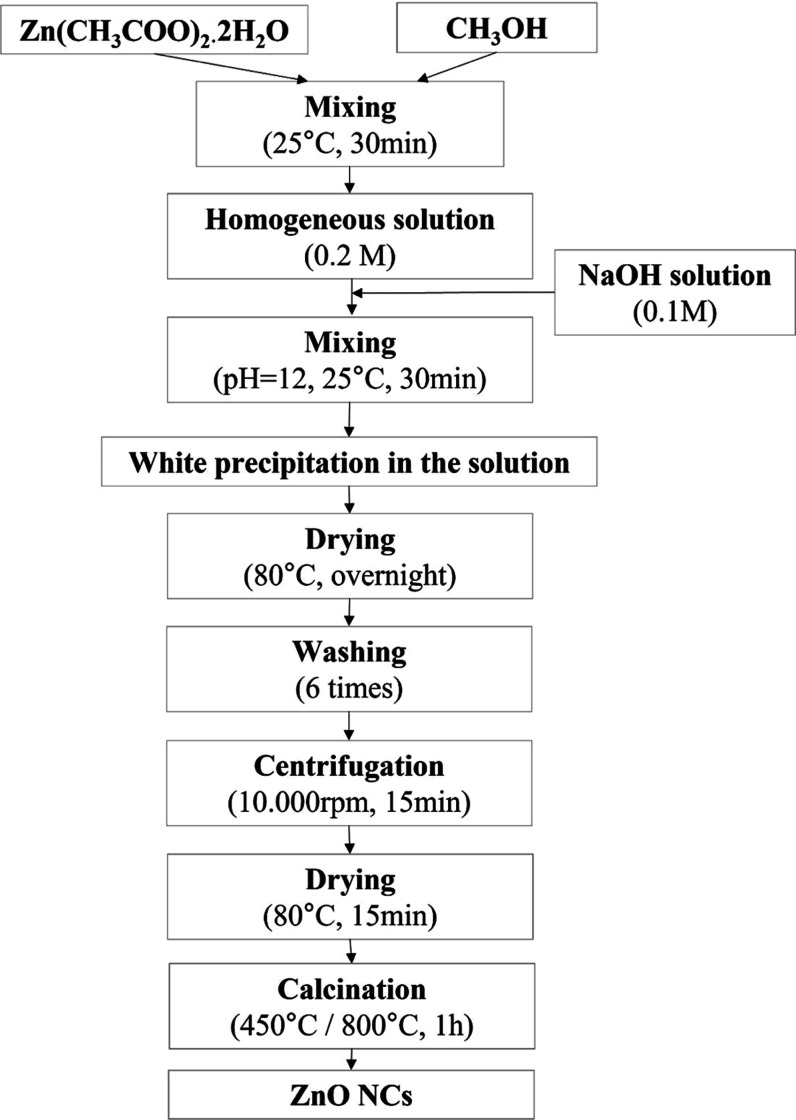
Flowchart of sol–gel synthesized ZnO NCs.

ZnO NCs were synthesized by the sol–gel method at pH = 12. To examine the effects of different calcination temperatures, two distinct temperatures, 800 and 450 °C, were utilized, and the resulting samples were designated as ZnO800 and ZnO450, respectively. Flow diagram of the synthesis steps of ZnO NCs using sol–gel are shown in Figure 1. First, zinc acetate dehydrate (Zn(CH_3_COO)_2_·2H_2_O) powder was dissolved in 100 mL of methanol (CH_3_OH) to give a concentration of 0.2 M.^29^ The homogeneous ZnO solution was obtained after stirring in a magnetic stirrer for 30 min. Separately, a homogeneous sodium hydroxide (NaOH) solution with a concentration of 1.0 M was prepared by dissolving 6 g of sodium hydroxide in 150 mL of deionized water after stirring at 25 °C for 30 min. 1.0 M NaOH solution was added dropwise to the ZnO solution to adjust the pH to 12. The solution turned milky white and was stirred in a magnetic stirrer for 1 h at constant room temperature with constant stirring. Considering the relationship between pH and precipitation time, the solution was dried in a magnetic stirrer at 80 °C overnight to obtain a white precipitation. The resulting precipitate was washed with methanol 6 times. It was then centrifuged at 10.000 rpm for 15 min to separate the white precipitate. After centrifugation, the resulting white, creamy sample was dried in a magnetic stirrer at 80 °C for 15 min. Finally, calcination was performed at 800 °C for 1 h to obtain ZnO800 NCs and at 450 °C for 1 h to obtain ZnO450 NCs.

### 2.3 Characterization of ZnO NCs

The crystal structure and average crystal size of ZnO NCs were determined by using X-ray diffraction (XRD) with Cu K_α_ radiation. The particle size, distribution, morphology, and shape of the ZnO NCs were characterized by using a field emission scanning electron microscope (FESEM). The hydrodynamic particle size distribution and zeta potential values of the ZnO NCs were measured. The optical reflectance spectra and band gap energies of the ZnO NCs were analyzed by using a UV–vis–NIR spectrophotometer.

### 2.4 HepG2 and HT29 Cell Culture

The HepG2 (ATCC HB-8065) hepatocellular carcinoma cell line and the HT29 (ATCC HTB-38) colorectal cancer cell line were commercially purchased from the American Type Culture Collection (ATCC) (Manassas, USA) for use in the study. For cell culture studies, the culture medium containing Dulbecco’s modified Eagle’s medium with 10% fetal bovine serum and penicillin/streptomycin (100 μg/mL; Gibco, USA) was prepared under sterile conditions and added to the cells. The flasks were incubated at 37 °C with 5% carbon dioxide in a humidified incubator.

### 2.5 3-(4,5-Dimethylthiazol-2-yl)–2,5-diphenyltetrazolium Bromide (MTT) Assay

Solutions of ZnO800 and ZnO450 were prepared mechanically in DMSO. ZnO NCs were added to HepG2 and HT29 cells at different concentrations of 250, 100, 50, 25, 5, and 1 μg/mL, and cells were incubated for 48 h. Control cells were treated with a culture medium only.

The MTT (3-(4,5-dimethyldiazol-2-yl)-2,5 diphenyl tetrazolium bromide) assay was used to determine the percentage of viable cells in the cell population by measuring the conversion of the yellow MTT dye to a dark blue-violet formazan product. To determine the IC_50_ values of ZnO NCs in HepG2 and HT29 cells, the method described by Yerlikaya et al. was used.^30^ Cells were grown in flasks and seeded in 96-well microplates at a density of 5000 cells/200 μL medium 24 h before the experiment. The different concentrations of ZnO NCs were applied to the cells and incubated for 48 h. Control cells were treated with culture medium only. Data were analyzed and plotted using GraphPad Prism 5.0 program (GraphPad Software, Inc., La Jolla, CA, USA). The data were normalized by nonlinear regression analysis using GraphPad Prism 5.0 to calculate IC_50_ values.

### 2.6 Cell Viability Analysis

The viability rates of the cells after treatment with ZnO800 and ZnO450 were calculated in comparison to the untreated control cells. The viability of the untreated cells was considered 100%, and the percent viability of the cells was calculated as follows
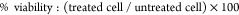
1

### 2.7 Oxidative Stress Analysis in HepG2 and HT29 Cells

Total antioxidant status (TAS) and total oxidant status (TOS) were determined according to the protocol using commercial kits (Rel Assay Diagnostics, Turkey).^31,32^ The ratio TOS/TAS is considered as the oxidative stress index (OSI). MDA content was quantified in the tissue and cell samples using the thiobarbituric acid reaction assay according to the method described by Ledwozyw et al.^33^ Absorbance was measured at 535 nm, and concentration was expressed as nmol MDA/g protein. The enzyme activity of catalase (CAT) was measured by the decrease in absorbance at 240 nm due to the consumption of hydrogen peroxide (H_2_O_2_).^34^ Enzyme activity was expressed as U/mg of protein. Total GSH content was determined by the dithionitrobenzoic acid (DTNB) recycling method.^35^ GSH concentration was expressed as nanomoles of GSH/g of protein. Total protein was determined by the method of Bradford.^36^ The intensity of blue development was measured at 595 nm against the blank. Total protein concentration was expressed in μg/μL. CAT, MDA, and total GSH values were calculated by normalizing them to the total protein values.

### 2.8 Antimicrobial Activity

ZnO NCs solutions were tested to determine the minimum inhibitory concentration (MIC), minimum bactericidal concentration (MBC), and minimum fungicidal concentration (MFC). The tested organisms included the Gram-positive bacteria *S. aureus* (ATCC 29213) and *Enterococcus faecalis* (ATCC 29212), the Gram-negative bacteria *P. aeruginosa* (ATCC 277853) and *E. coli* (ATCC 25922), and *Candida parapsilopsis* (ATCC 22019) as a yeast. These microorganisms are known to have a pathogenic effect on humans. Chloramphenicol (10 mg/mL) and Bacitracin (10 mg/mL) for bacteria and Ketoconazole (25 mg/mL) for yeast were used as control drugs. The microdilution method was performed according to the Clinical and Laboratory Standards Institute.^37,38^

The stock solutions of ZnO800 and ZnO450 were prepared at a concentration of 2.5 mg/mL. Dilutions were made from the ZnO800 stock solution to achieve concentrations of 250, 131 (IC_50_ value to HT29), 100, 50, 25, 5.74 (IC_50_ value to HepG2), 5, and 1 μg/mL. Likewise, dilutions were made from the ZnO450 stock solution to obtain 250, 122 (IC_50_ value to HT29), 100, 50, 26 (IC_50_ value to HepG2), 25, 5, and 1 μg/mL. The dilutions of ZnO NCs were prepared in MHB and SDB. 100 μL of the different dilutions of ZnO NCs were added to the wells. In each well, 100 μL of the overnight culture, adjusted to a 0.5 McFarland standard, was added. The bacterial-inoculated plates were incubated at 37 °C for 24 h, while the yeast-inoculated plates were incubated at 28 °C for 72 h. After incubation, the lowest concentration without growth was determined as the MIC value. The plates were inoculated with MHA for bacteria and SDA for yeast at various concentrations to determine the lowest concentration that showed no bacterial or yeast growth. After incubation, the plates were examined to see if there was any growth. The lowest concentration without any growth was recorded as the MBC for bacteria or the MFC for yeast. The experiments were performed three times. Growth was controlled with tetrazolium chloride (TCC).

## 3 Results and Discussion

### 3.1 XRD Analysis of ZnO NCs

**Figure 2:**
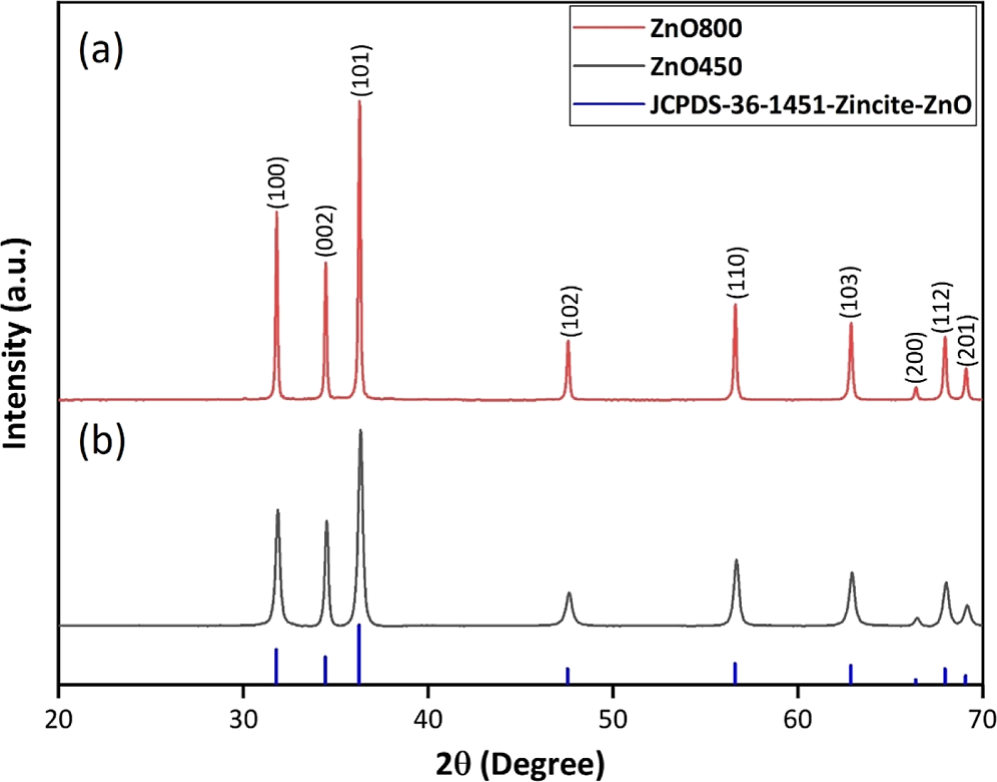
XRD patterns of (a) ZnO800 and (b) ZnO450.

XRD was employed to investigate and characterize the crystalline behavior of ZnO NCs. The examination revealed the typical wurtzite hexagonal ZnO structure for both ZnO800 and ZnO450 samples (JCPDS 00-036-1451) in Figure 2. The data were taken with Cu Kα radiation (1 = 1.5056 Å) in the range of 20° ≤ 2θ ≤ 70°. No additional peaks related to other phases were detected, indicating the production of high-purity ZnO NCs. These findings are consistent with other recent studies on ZnO nanoparticles using the sol–gel method.^39−43^ The main peak at a calcination temperature of 800 °C is sharper and has a smaller full width at half-maximum (fwhm) compared to 450 °C (Figure 2a,b).

The crystallite sizes of ZnO NCs were determined using the Scherrer’s formula, which utilizes the fwhm of the peak observed in XRD. This measurement relates the crystal size, as described by the Scherrer equation
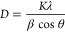
2where *D* is the average crystallite size of the phase under investigation, *K* is the Scherrer constant (0.89), λ is the wavelength length of the radiation used (0.15406 nm), β is the fwhm of diffraction (in radians), and θ is peak position of diffraction (in radians).

The crystal sizes were determined by analyzing various peaks in the diffraction patterns. The average crystallite size was found to be 52.65 nm for ZnO800 and 25.11 nm for ZnO450. Similar average crystal sizes of 26.1 nm were reported by Sahai and Goswami, who annealed ZnO at 400 °C for 3 h, and Singh et al., who obtained an average crystal size of 26 nm for ZnO nanoparticles by annealing at 500 °C for 8 h.^40,44^ Compared to a calcination temperature of 800 °C, subjecting the NCs to a 1 h calcination at 450 °C resulted in a reduction in the average crystal size. This finding demonstrates the feasibility of synthesizing NCs with smaller crystallite sizes through the use of lower calcination temperatures and shorter processing times. Increasing the sintering temperature promotes the growth of crystals, leading to an increase in the crystallite size. This phenomenon can be attributed to enhanced diffusion and atomic mobility at higher temperatures, allowing atoms to migrate and coalesce, resulting in larger crystal structures.

### 3.2 SEM Analysis of ZnO NCs

**Figure 3:**
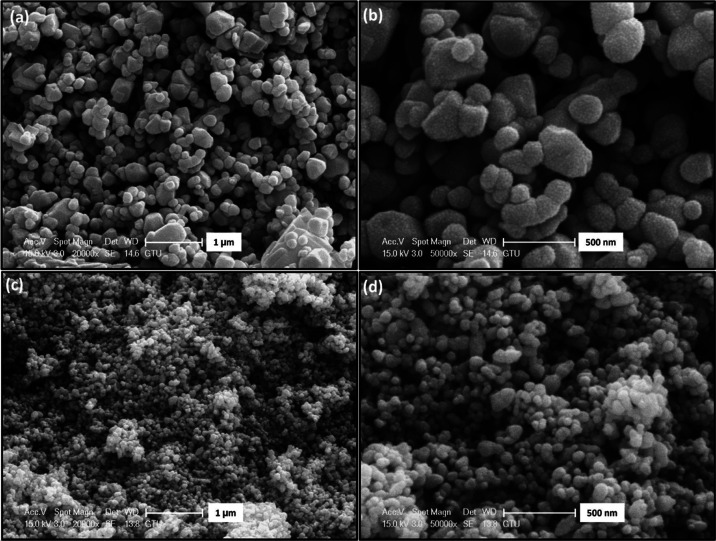
SEM images of ZnO NCs synthesized by sol–gel method: (a) at low and (b) at high magnification of ZnO800 and (c) at low and (d) at high magnification of ZnO450.

Figure 3a,b depicts the surface morphology of ZnO800 and ZnO450 zinc oxide nanoparticles (ZnO NCs) synthesized using the sol–gel method. The micrograph reveals that ZnO800 NCs exhibit subangular grains with a combination of rounded and sharp edges, representing a morphology that lies between equiaxed and angular grains. The grain size distribution observed in the micrograph appears random and heterogeneous, indicating nonuniform processing conditions and uneven nucleation without a discernible pattern. The grain sizes ranged from 100 to 500 nm. In contrast, ZnO450 NCs exhibit predominantly spherical particles with grain sizes below 100 nm. In both SEM images, the particles are observed to form bundles due to agglomeration. As the calcination temperature is decreased to 450 °C, the degree of agglomeration decreases.

### 3.3 Particle Size Distribution and Zeta Potentials of ZnO NCs

**Figure 4:**
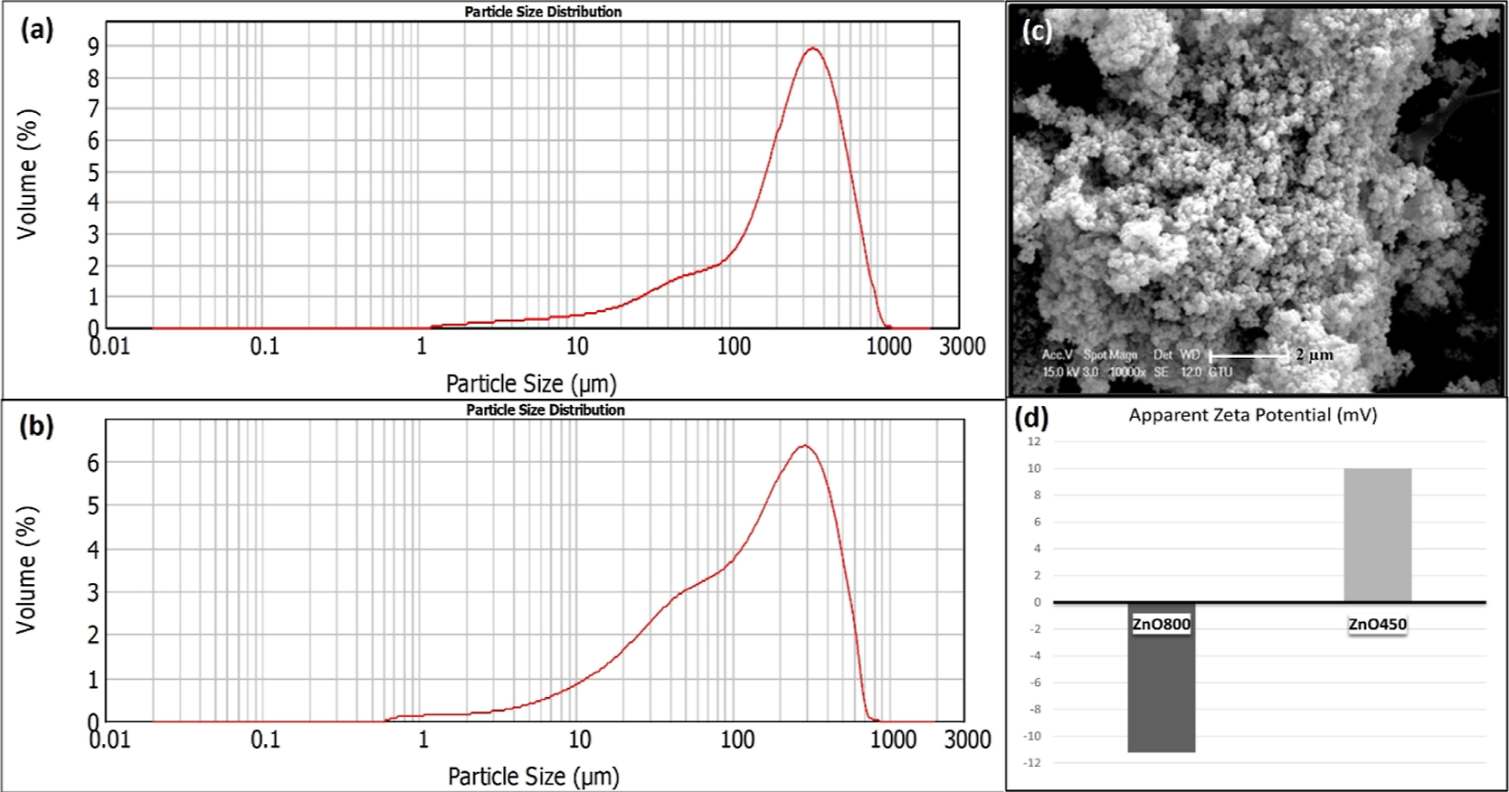
Hydrodynamic particle size distribution of (a) ZnO800, (b) ZnO450, (c) SEM image of agglomerated nanoparticles, and (d) zeta potentials of ZnO NCs.

Figure 4 shows the particle size distributions of ZnO800 (a) and ZnO450 (b), determined by the laser diffraction method. The particle size distribution graphs show oscillating patterns, indicating the predominant agglomeration of the particles of ZnO NCs. This observation is consistent with the limitations of the light diffraction technique, which tends to emphasize larger particles and thereby overshadow the visibility of monodisperse, smaller particles in the sample.^45^Table 1 compares the average crystal sizes, grain sizes, and hydrodynamic particle size distributions, including volume moment mean (*D*_[4,3]_), surface area mean (*D*_[3,2]_), and percentile values of the distribution. The surface area mean (Sauter mean diameter) is critical for surface-related properties such as bioavailability and dissolution, as it is very sensitive to fine particles in the distribution.^46^ The surface area mean of the ZnO800 and ZnO450 particles was 69.30 and 34.56 μm, respectively, indicating a larger surface area mean for ZnO450. Figure 4c shows the SEM image of aggregated and accumulated particles, which explains why the average particle sizes measured by digital light scattering were significantly larger than typically expected for nanoparticles due to particle agglomeration. Figure 4d shows the zeta potential of the ZnO NCs. A notable difference is that ZnO800 has a zeta potential of −11.2 mV, indicating a relatively stable colloidal dispersion, while ZnO450 has a zeta potential of +10 mV.

**Table 1:** Crystal Size, Grain Size, Particle Size Distribution, and Zeta Potential Values of ZnO NCs

			particle size distribution (μm)					
	XRD crystal size (nm)	SEM grain size (nm)	the volume moment mean *D*_[4,3]_	the surface area mean *D*_[3,2]_	*d*^(0.1)^	*d*^(0.5)^	*d*^(0.9)^	zeta potential (mV)
ZnO800	52.65	100–500	288.42	69.30	42.13	265.38	563.74	–11.2
ZnO450	25.11	<100	189.11	34.56	19.24	148.29	428.48	10

### 3.4 UV–vis–NIR Spectroscopy Analysis of ZnO NCs

**Figure 5:**
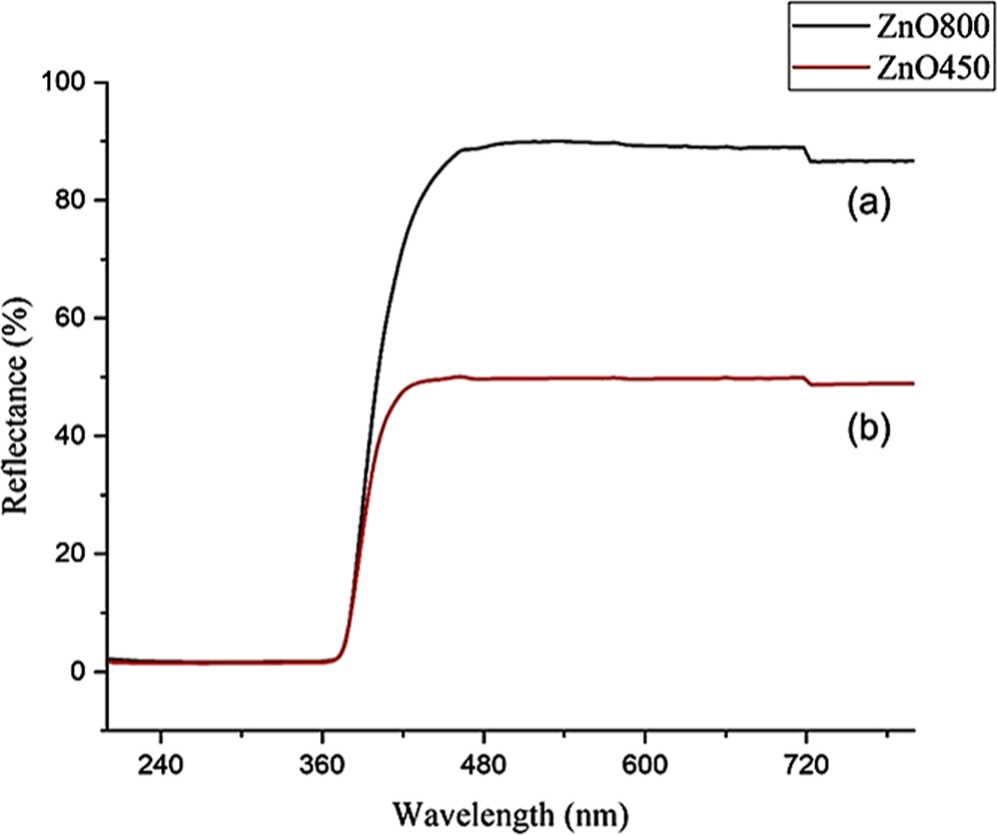
Reflectance spectra of ZnO NCs calcined at (a) 800 °C and (b) 450 °C.

The UV–vis reflectance spectrum of ZnO nanoparticles reveals a distinctive band gap absorption in the UV region (below 400 nm), reflecting the energy needed for electronic transitions from the valence band to the conduction band, confirming ZnO’s semiconductor characteristics. The reflectance spectra showed a sharp increase around 370 nm for both ZnO800 and ZnO450 in Figure 5a,b. The reflectance percentage in the visible range was around 90 and 50% for ZnO800 and ZnO450, respectively (Figure 5a,b). The high reflectance values indicate that a significant portion of incident light is reflected back from the surface of the nanoparticles at the corresponding wavelengths. Factors such as particle size, morphology, and surface roughness can influence the reflectance properties of the nanoparticles and lead to variations in the observed reflectance percentages. ZnO450 has a lower reflectance since, at smaller sizes, the nanoparticles have a larger surface-to-volume ratio, which leads to increased light scattering and absorption. This enhanced interaction with light results in a reduced reflectance as more light is absorbed or scattered away from the surface of the nanoparticles. The lower reflectance of ZnO-450, which indicates higher light absorption, makes it more suitable for therapies such as photodynamic cancer therapy, where increased light absorption can increase the production of ROS to attack cancer cells. Research by Lestari et al. has shown that ZnO nanoparticles, especially in combination with UV radiation, significantly reduce the viability of MCF-7 breast cancer cells.^47^ The study underlines the effective use of ZnO nanoparticles as a potential anticancer agent, especially in combination with UV irradiation. In addition, this property may improve its efficacy in antimicrobial and antioxidant applications as higher light absorption may lead to more effective ROS-mediated microbial inhibition and neutralization of harmful free radicals.

The band gap energy of ZnO NCs was determined using UV–vis–NIR spectrophotometry and the Tauc relation. The measured band gap values for ZnO800 and ZnO450 were 3.26 and 3.28 eV, respectively. These results indicate that the band gap slightly increases with decreasing annealing temperature. When comparing ZnO800 and ZnO450 with the bulk form of ZnO, which has a band gap of 3.37 eV, there are several factors that contribute to the difference in the band gap between ZnO powder and ZnO in its bulk form. ZnO powder may contain a higher concentration of crystal defects, such as vacancies, which introduce localized states within the band gap and affect its effective value. Additionally, the surface of ZnO powder exhibits a higher proportion of atoms with unsatisfied bonds, leading to surface states that interact with the electronic states in the band gap and modify its effective value. Furthermore, ZnO powder typically consists of particles with a range of sizes including smaller nanoparticles. As the particle size decreases, the quantum confinement effect becomes more pronounced, resulting in changes in the electronic structure and band gap compared to those of the bulk material. Overall, the combination of crystal defects, surface effects, and particle size distribution contributes to the observed difference in the band gap between ZnO powder and ZnO bulk. The large band gap of ZnO nanoparticles (3.37 eV) is a limitation for applications in areas such as anticancer, antimicrobial, and antioxidant therapies due to its influence on ROS generation and cell viability. Lowering the band gap by increasing the surface area can enhance the nanoparticles’ interaction with light, thereby improving ROS generation. This modification could lead to more effective treatments in cancer therapy, as well as enhanced antimicrobial and antioxidant activities, making ZnO nanoparticles more practical and versatile for various medical applications.^48^

### 3.5 Cytotoxic Effects of ZnO800 and ZnO450 NCs on HepG2 and HT29

The present study aimed to evaluate the potential of ZnO NCs as anticancer agents by investigating their cytotoxic effects on HepG2 and HT29 cells. HepG2 and HT29 cells were cultured in standard growth medium for 24 h before being treated with ZnO800 and ZnO450 NCs. Various concentrations of ZnO800 and ZnO450 NCs (250, 100, 50, 25, 5, and 1 μg/mL) were applied to the cells and incubated for 48 h. Control cells were treated with the culture medium alone. After the treatment period, the MTT assay was conducted on HepG2 and HT29 cells to determine the cell viability. Statistical analysis revealed IC_50_ values of 5.74 and 26 μg/mL for HepG2 cells treated with ZnO800 and ZnO450 NCs, respectively. Similarly, IC_50_ values of 131 and 122 μg/mL were obtained for HT29 cells treated with ZnO800 and ZnO450 NCs, respectively. The results suggest that HepG2 cells demonstrated higher sensitivity to the cytotoxic effects of both types of NCs compared with HT29 cells. The study conducted by Sevki et al. further supports these results, reporting IC_50_ values of 33.9 μg/mL for HepG2 cells and 38.6 μg/mL for HT29 cells when treated with ZnO nanoparticles. These variations in IC_50_ values among studies can be attributed to factors such as nanoparticle size, concentration, exposure time, and assay methods. Overall, these findings highlight the cytotoxic potential of ZnO nanoparticles on both HepG2 and HT29 cells, with HepG2 cells demonstrating a higher sensitivity to their cytotoxic effects.

To assess the cell survival rate of HepG2 and HT29 cells treated with varying concentrations of ZnO800 and ZnO450 NCs, the cells were cultured in 96-well microplates until they reached the logarithmic growth phase. Subsequently, HepG2 and HT29 cells were exposed to concentrations of 250, 100, 50, 25, 5, and 1 μg/mL of ZnO800 and ZnO450 NCs for a duration of 48 h.

**Figure 6:**
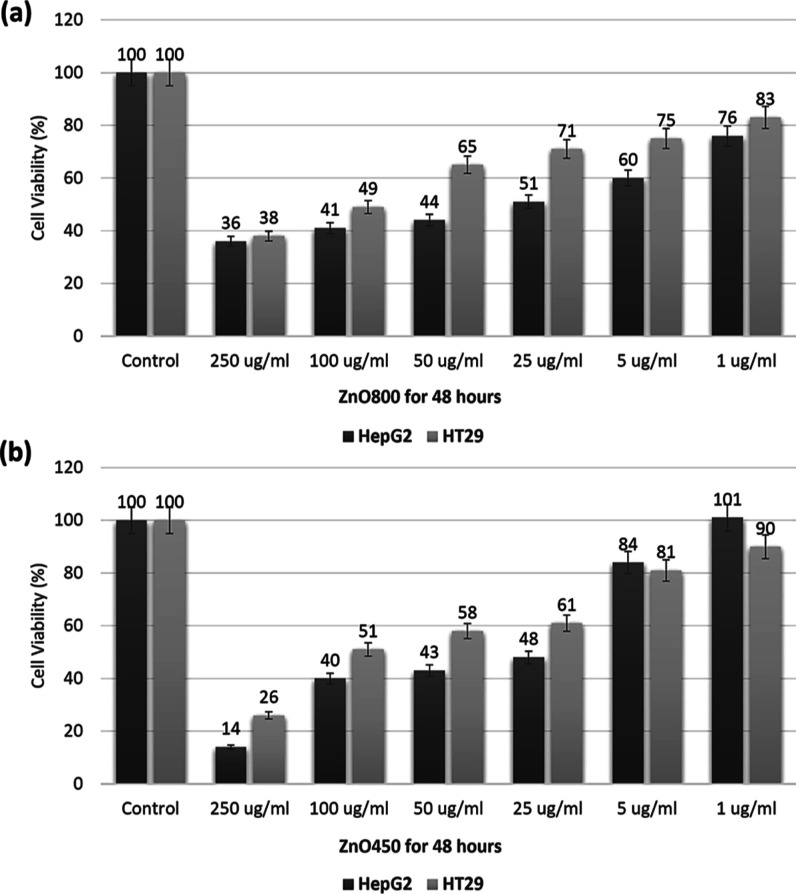
HepG2 and HT29 cell viability (%) after (a) ZnO800 and (b) ZnO450 treatment for 48 h.

The viability of HepG2 cells at higher ZnO800 concentrations was measured at 36, 41, 44, and 51%, respectively. However, as the concentration decreased to 5 and 1 μg/mL, the cell viability increased to 60 and 76%, respectively. Similarly, HT29 cells exhibited viabilities of 38, 49, 65, and 71% at the higher ZnO800 concentrations, with viabilities rising to 75 and 83% at the lower concentrations (as depicted in Figure 6a). These results indicate that both HepG2 and HT29 cells demonstrated lower viability compared to the control group, but the viability increased when the ZnO800 concentration decreased to 1 μg/mL. Furthermore, ZnO800 treatment had a more pronounced impact on reducing viability in HepG2 cells, suggesting their greater sensitivity to ZnO800 nanoparticles compared to HT29 cells.

In HepG2 cells, the viability was 14, 40, 43, and 48% at higher concentrations of ZnO450 NCs. However, at lower concentrations, the viability increased to 84% and even surpassed that of the control group, reaching 101%. Similarly, for HT29 cells, the viability at the higher concentrations of ZnO450 NCs was 26, 51, 58, and 61%, but it increased to 81 and 90% at the lower concentrations, as depicted in Figure 6b. These findings demonstrate a dose-dependent response, wherein higher concentrations of ZnO450 NCs exhibit cytotoxic effects, resulting in a decreased cell viability. However, at lower concentrations, the cells may have exhibited some degree of adaptation or resistance, leading to increased viability in comparison with the control group. Furthermore, the lowest viability rates were observed in HepG2 and HT29 cells, with respective rates of 14 and 26% at a concentration of 250 μg/mL for ZnO450, as compared to the same concentration of ZnO800.

### 3.6 Effects of ZnO800 and ZnO450 on Oxidative Stress Parameters in HepG2 and HT29 Cancer Cells

The IC_50_ values of ZnO800 and ZnO450 were selected as the concentrations of ZnO NCs for determination of the oxidative stress parameters. Specifically, the IC_50_ values for HepG2 cells were 5.74 and 26 μg/mL, corresponding to the concentrations of ZnO800 and ZnO450, respectively, whereas IC_50_ values for HT29 cells were 131 and 122 μg/mL corresponding to the concentrations of ZnO800 and ZnO450, respectively. The effects of ZnO800 and ZnO450 on the TAS, TOS level, catalase enzyme activity (CAT), total glutathione (GSH) levels, and malondialdehyde (MDA) levels were investigated, revealing the relationship between oxidative stress, antioxidant capacity, and the activity of key antioxidant parameters.

**Figure 7:**
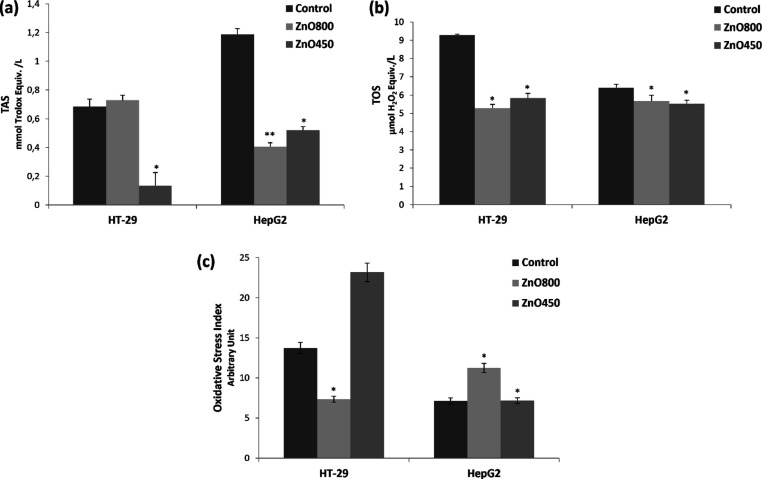
(a) TAS in HT-29 and HepG2 cells after exposure to ZnO800 and ZnO450. Results are given as a mean + standard error. *p < 0.05, **p < 0.01, (b) TOS in HT-29 and HepG2 cells, after exposed to ZnO800 and ZnO450. Results are given as a mean + standard error. *p < 0.05, **p < 0.01, (c) OSI in HT-29 and HepG2 cells, after exposed to ZnO800 and ZnO450. Results are given as a mean + standard error. *p < 0.05, **p < 0.01.

In the HT-29 cell line, ZnO450 significantly reduced TAS levels, indicating a decrease in antioxidant capacity. On the other hand, the visible change resulting from ZnO800 application in the HT-29 cell line was not statistically significant, suggesting that it did not significantly affect TAS levels. In the HepG2 cell line, both forms of ZnO NCs (ZnO800 and ZnO450) resulted in a decrease in TAS levels, indicating a reduction in antioxidant capacity (Figure 7a).

In both the HT-29 cell line and the HepG2 cell line, both forms of ZnO NCs (ZnO800 and ZnO450) led to a significant decrease compared to the control in the TOS. This indicates that the application of ZnO800 and ZnO450 resulted in a reduction in the overall oxidative stress in both cell lines (Figure 7b). On the other hand, the TOS values of both ZnO NCs in both cell lines are increasing compared with the TAS values. The OSI was calculated to estimate the balance between oxidants and antioxidants and to serve as an indicator of oxidative stress. The OSI is calculated according to the following equation
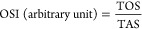
3

There was an increase in OSI that indicates an elevation in oxidative stress except for ZnO800 in HT29 cells (Figure 7c).

**Figure 8:**
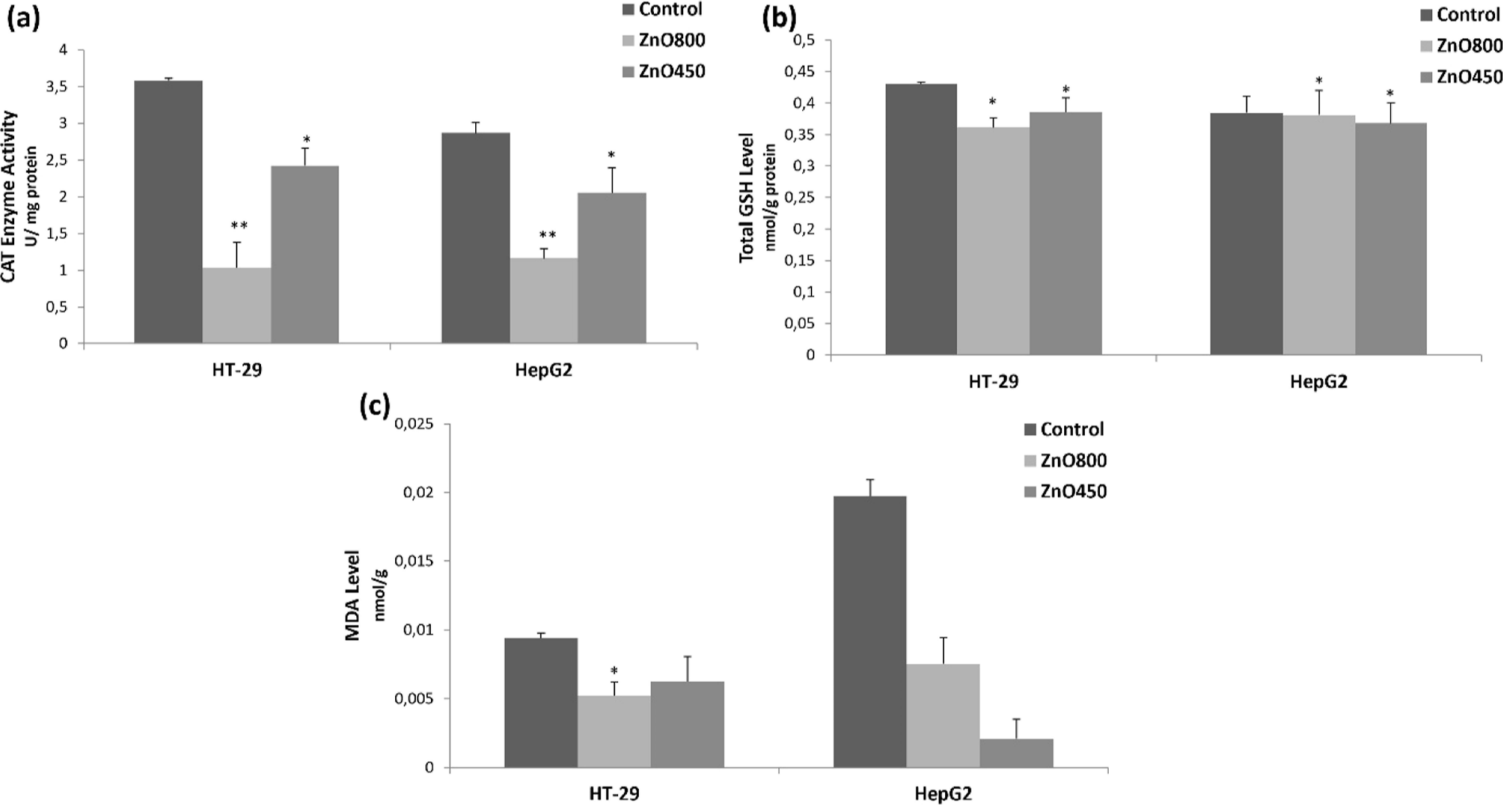
(a) CAT enzyme activity in HT-29 and HepG2 cells, after exposure to ZnO-1 and ZnO-2. Results are given as a mean + standard error. *p < 0.05, **p < 0.01, (b) total glutathione (GSH) level in HT-29 and HepG2 cells, after exposed to ZnO-1 and ZnO-2. Results are given as a mean + standard error. *p < 0.05, **p < 0.01, (c) MDA level in HT-29 and HepG2 cells, after exposed to ZnO-1 and ZnO-2. Results are given as a mean + standard error. *p < 0.05, **p < 0.01.

The activity of enzyme CAT showed a significant decrease in both HT-29 and HepG2 cell lines after the administration of ZnO800 and ZnO450 NCs, as depicted in Figure 8a. In addition, the total glutathione (GSH) level demonstrated a statistically significant decrease in HT-29 cells upon exposure to ZnO800 and ZnO450, while there was no significant change observed in the ZnO800 and ZnO450 groups of HepG2 cells, as shown in Figure 8b. Furthermore, the level of MDA, which is indicative of oxidative stress, significantly decreased in both HT-29 and HepG2 cell lines following the administration of ZnO800 and ZnO450 NCs, as illustrated in Figure 8c. It is important to note that the MDA level is correlated with TOS levels.

The main aim of this part of the study was to examine how the crystal size of nanoparticles affects their antioxidant effects. The results showed that ZnO450 nanoparticles with a size of 25.11 nm were more effective in HT29 cells, while ZnO800 nanoparticles with a size of 52.65 nm had a stronger impact on HepG2 cells. These findings suggest that the varying crystal sizes of the nanoparticles can influence their antioxidant activity. It is worth noting that previous studies have reported significant antioxidant activity in spherical ZnO nanoparticles with a size of 32 nm,^49^ and an increase in antioxidant activity with higher concentrations of ZnO nanoparticles with an average size of 46.49 nm.^50^ Studies have shown that ZnO nanoparticles induce apoptotic death in human breast (MCF7) and colon cancer (HT29) cells by weakening the antioxidant defense system, which in turn increases ROS levels.^51,52^ It is possible that ZnO450 NCs, when closer to the optimal size for cellular uptake, have a more effective cytotoxic effect on HT29 cells compared to ZnO800 NCs in Figure 7c. Studies have also focused on how ZnO nanoparticles affect the cell cycle regulation and induction of apoptosis in various human cancers. The interplay between apoptosis induction and cell cycle regulation is critical as failure to induce an apoptotic response can lead to uncontrolled cell proliferation and cancer development. Key strategies include the control of oxidative stress and the promotion of early, safe cell death, particularly through the activation of P53 and BAX. P53 plays a crucial role in responding to DNA damage, regulating the cell cycle, and triggering transcription of the pro-apoptotic BAX gene, which leads to apoptosis. The increase in BAX expression counteracts the antiapoptotic effects of the BCL2 protein and thus facilitates apoptosis and natural cell death. Conversely, the BCL2 protein inhibits the apoptosis pathway and thus contributes to the development of cancer phenotypes.^51^ This phenomenon may also have occurred with ZnO800 NCs for the HT29 cells. Further research is necessary to understand the underlying mechanisms and to explore the potential applications of these nanoparticles in conditions associated with oxidative stress.

### 3.7 Antimicrobial Activity of ZnO NCs

The antimicrobial susceptibility of ZnO NCs was evaluated against various microorganisms, including *E. faecalis*, *S. aureus*, *E. coli*, *P. aeruginosa*, and *C. parapsilopsis*, using the broth microdilution method. The MIC values of ZnO NCs are presented in Table 2. For ZnO800, the MIC values were determined as 1, 25, 131, and 5.74 μg/mL against *E. faecalis*, *S. aureus*, *P. aeruginosa*, and *C. parapsilopsis*, respectively. ZnO450 exhibited MIC values of 1, 26, and 25 μg/mL against *E. faecalis*, *S. aureus*, and *C. parapsilopsis*, respectively, indicating the effectiveness of both ZnO NCs, with ZnO450 showing greater efficacy at lower concentrations. The antimicrobial mechanisms of ZnO NCs are closely linked to their physical properties, as various studies have shown. For example, Jiang et al. observed that ZnO nanoparticles with an average size of about 30 nm were effective against *E. coli* and caused cell death by directly destroying the phospholipid bilayer of the bacterial membrane. The important role of ROS in the antibacterial properties of these nanoparticles was emphasized by the finding that free radical scavengers such as mannitol, vitamin E and glutathione were able to inhibit the bactericidal effect of the ZnO nanoparticles.^53^ In a separate study, Reddy et al. synthesized ZnO nanoparticles with a size of approximately 13 nm and demonstrated their pronounced antibacterial activities against *E. coli*, effectively stopping its growth at concentrations around 3.4 mM, and against *S. aureus* at lower concentrations, starting at 1 mM.^54^ To further substantiate these results, Ohira and co-workers discovered that the antibacterial activity of ZnO nanoparticles against both *E. coli* and *S. aureus* was more pronounced at smaller crystallite sizes. This was attributed to the greater amount of Zn^2+^ ions released by the smaller ZnO nanoparticles compared to larger ZnO particles, with *E. coli* being more sensitive to Zn^2+^ ions than *S. aureus*. Overall, these studies emphasize the key role of Zn^2+^ ions and the influence of nanoparticle size on the antibacterial efficacy of ZnO nanoparticles.^55^

**Table 2:** MIC and MBC/MFC Values of ZnO NCs

microorganisms		Gram-positive bacteria		Gram-negative bacteria		fungi
		E. faecalis	S. aureus	E. coli	P. aeruginosa	C. parapsilopsis
MIC values (μg/mL)	ZnO800	1	25	—	131	5,74
	ZnO450	1	26	—		25
MBC/MFC values (μg/mL)	ZnO800	5,74	131	—	250	50
	ZnO450	25	122	—	—	50
Chloramphenicol (10 mg/mL) and Bacitracin (10 mg/mL)/Ketoconazole (25 mg/mL)	—	—	—	—	—	—
(+)-control	+	+	+	+	+	+

The antimicrobial activity of ZnO NCs can be attributed to the production of free radicals and the induction of oxidative stress, which disrupts the bacterial membrane.^56^ These results also demonstrate the greater effectiveness of ZnO NCs against Gram-positive bacteria compared with Gram-negative bacteria. Among the tested microorganisms, *E. coli* was the most resistant, while *E. faecalis* showed the highest sensitivity to ZnO NCs, displaying bactericidal activity even at low doses (5.74 μg/mL for ZnO800, 25 μg/mL for ZnO450). Previous research has consistently shown the antibacterial activity of ZnO NCs against *S. aureus* and *E. coli*, with *S. aureus* being more sensitive.^57,58^ The differences in the susceptibility of microorganisms to antibacterial agents such as ZnO nanoparticles can be quite pronounced, as shown by the higher susceptibility of *E. faecalis* compared to *E. coli*. These differences are often due to the inherent structural and physiological differences between the organisms. *E. faecalis*, a Gram-positive bacterium, has a relatively simpler cell wall structure with a thick peptidoglycan layer. This characteristic may make it more susceptible to the penetration and action of antibacterial agents, such as ZnO nanoparticles. The mechanism by which ZnO nanoparticles exert their antibacterial effect, possibly by generating ROS and disrupting cellular processes, may be more effective against the less complex cell wall of Gram-positive bacteria. In contrast, *E. coli*, a Gram-negative bacterium, has a more complex outer membrane rich in lipopolysaccharides that can act as a barrier to antibacterial agents.^59^ In addition, as reported in other studies, *E. coli* has the ability to develop adaptive resistance to ZnO nanoparticles. This adaptation mechanism is characterized by changes in the bacterial morphology and expression of membrane proteins. Such adaptations may allow *E. coli* to temporarily resist the antibacterial effect of ZnO nanoparticles, although this resistance is not permanent and can be reversed after cessation of exposure. The development of this reversible resistance suggests that *E. coli* may activate defense mechanisms that temporarily protect it from the effects of ZnO nanoparticles, which may not be as pronounced or effective in Gram-positive bacteria such as *E. faecalis*. In further studies, the resistance of *E. coli* and other Gram-negative bacterial species can be investigated when the ZnO nanoparticles are exposed over a longer period of time to study the effect of ZnO nanoparticles on Gram-negative bacteria. Understanding these mechanisms is crucial for the development of more effective antibacterial strategies and prediction of changes in bacterial susceptibility patterns over time. While the phenomenon of bacterial nanoresistance is still not fully understood, it is a critical factor in the ongoing development of nanoparticle-based antibacterial applications.^60^

In addition to the bacterial studies, this research also investigated the antifungal activity of ZnO NCs against *C. parapsilopsis*. The results demonstrated the sensitivity of *C. parapsilopsis* to ZnO NCs, highlighting their antifungal potential. Overall, these findings underscore the broad antimicrobial efficacy of ZnO NCs, particularly against Gram-positive bacteria and the fungus *C. parapsilopsis*.

This study on ZnO NCs investigated their potential for cancer therapy, focusing on the crystalline behavior, surface morphology, particle size and zeta potential of ZnO800 and ZnO450 NCs. It emphasized the influence of calcination temperature on crystal size, which is crucial for biological interactions and therapeutic applications. The research also demonstrated their cytotoxic effect on HepG2 and HT29 cells as well as their antimicrobial and antifungal activities, suggesting significant medical applications. In addition, the study investigated their optical properties, highlighting their potential for photodynamic cancer therapy. This comprehensive analysis highlights the need for further research to fully exploit the therapeutic potential of the ZnO NCs.

## 4 Conclusions

This comprehensive study analyzes the structural, optical, and biological properties of ZnO NCs. XRD confirmed the wurtzite hexagonal structure of ZnO800 and ZnO450 with a crystallite size of 52.65 and 25.11 nm, respectively. Morphological analysis revealed subangular and spherical grains for ZnO800 and ZnO450. In addition, the particle size distribution and zeta potential of these ZnO NCs were thoroughly investigated. A notable finding is the higher light absorption observed in ZnO450, suggesting potential applications in photodynamic and antimicrobial therapies. Cytotoxicity assays have shown significant effects on the cancer cell lines HepG2 and HT29, with HepG2 cells exhibiting a higher sensitivity to ZnO NCs. In addition, both ZnO800 and ZnO450 have shown strong antimicrobial activity, particularly against Gram-positive bacteria and *Candida parapsilosis*, highlighting their potential as versatile therapeutic agents. Despite these promising results, the study acknowledges certain limitations. There is a need for in vivo testing to confirm the efficacy and safety of these NCs as well as a deeper investigation of the underlying mechanisms of action. Future research directions could include optimizing the sol–gel synthesis process for specific therapeutic applications, investigating targeted delivery methods, and exploring the interactions of these NCs with different types of cancer cells and microbial species. Such research efforts are critical to advance the development of more effective and targeted cancer therapies and antimicrobial strategies using ZnO NCs.
